# Petrographic image classification of complex carbonate rocks from the Brazilian pre-salt using convolutional neural networks

**DOI:** 10.1038/s41598-025-10006-0

**Published:** 2025-08-21

**Authors:** Mateus Basso, João Paulo da Ponte Souza, Guilherme Furlan Chinelatto, Luis Augusto Antoniossi Mansini, Alexandre Campane Vidal

**Affiliations:** 1 Reservoir Geological Modelling Lab, CEPETRO, 13083-896 Campinas, Brazil; 2 Geology and Natural Resources Department, Institute of Geosciences, 13083-855 Campinas, Brazil

**Keywords:** Machine learning, Thin section, Petrography, Brazilian pre-salt, Geology, Petrology, Sedimentology, Computational science

## Abstract

Machine learning (ML) algorithms have been widely applied across geosciences for tasks such as data conditioning, resolution enhancement, and image classification. The use of ML enables the analysis of large datasets, the identification of complex patterns, and can save time and reduce costs compared to conventional approaches. Among these techniques, Convolutional Neural Networks (CNNs) have emerged as powerful tools for image classification in various geoscientific applications. In the context of the carbonate reservoirs of the Brazilian Pre-salt, the sedimentological complexity of these deposits, combined with the vast amounts of data produced, drives the need for automated image classification approaches. Although several recent studies have explored ML methods for petrographic image analysis in diverse geological settings, few have focused specifically on the complex carbonates of the Brazilian Pre-salt reservoirs. In this study, we present a fully automated and modular machine learning workflow for petrographic image classification of thin sections from the Aptian Barra Velha Formation, Santos Basin, Brazil. Our approach includes the direct integration of paired plane-polarized light (PPL) and cross-polarized light (XPL) images as raw inputs to deep learning models, allowing for a more comprehensive representation of petrographic features. Additionally, we implement a hierarchical classification scheme, based on facies upscaling, encompassing three levels of classification granularity: a simplified scheme with 5 classes, an intermediate with 9 classes, and a complete scheme with 23 classes, a dimension not systematically explored in previous studies. Our dataset comprises 800 thin sections, corresponding to 1,600 high-resolution scanned images (6,400 dpi), from six wells across three different oilfields, strategically selected to ensure representativeness across distinct structural domains of the reservoir. We evaluated five computational models: EfficientNet, MobileNet v3, RegNet, ResNet, and ShuffleNet v2. The models MobileNet v3 large, RegNet x 800mf, and RegNet y 400mf achieved the highest F1-scores for the simplified (0.795), intermediate (0.768), and complete classifications (0.528), respectively. Notably, the intermediate classification with nine classes offered the best balance between detail and accuracy. This work presents a promising approach for automatic petrographic image pre-classification, favoring efficient database organization in the challenging exploratory settings of the Brazilian Pre-Salt.

## Introduction

Machine learning (ML) methods have gained widespread application across various branches of geosciences, driven by their capacity to address diverse and complex tasks. The integration of ML techniques allows for the processing and analysis of vast datasets, uncovering intricate patterns that are often not easily discernible through traditional approaches. Additionally, ML methodologies have the potential to streamline workflows, reduce manual labor, and cut operational costs by offering faster alternatives to conventional techniques^1–4^.

Originally developed for applications in computer vision, Convolutional Neural Networks (CNNs) have proven to be effective tools for image recognition and classification tasks across many scientific domains. In geosciences, several studies have demonstrated the utility of CNNs in a wide range of applications, from seismic interpretation to lithological classification^5–8^. CNNs have shown particular promise in the analysis of petrographic images, enabling the classification of minerals, textures, and other microstructural features in ways that enhance or complement manual petrographic techniques^9^.

A growing body of research has explored the potential of machine learning (ML) algorithms, including CNNs, for analyzing petrographic images across various geological contexts and objectives (e.g^9–13^.). These studies have significantly advanced the field; however, there remains scope for further development and application of such methods to address specific geological challenges, particularly those involving complex sedimentary systems. One prominent example is the unique geological context of Brazil’s pre-salt reservoirs, especially the intricate and heterogeneous carbonates of the Aptian Barra Velha Formation (BVF).

Among these contributions, the work of^14^ represents an important step by applying ML techniques to generate mineralogical maps and identify porosity in petrographic thin sections from pre-salt carbonate samples. Their approach, based on manual feature engineering and traditional classifiers (artificial neural networks and random forests), demonstrated the feasibility of automated mineralogical and porosity segmentation. Nonetheless, it focused primarily on mineralogical mapping rather than broader applications such as facies classification or the exploration of hierarchical lithological structures.

More recent studies, including those by^15–17^, and^18^, have further expanded the application of machine learning to pre-salt carbonate rocks, employing diverse architectures and data types. For example^15^, applied handcrafted texture features and autoencoders for classification, while^18^ explored Vision Transformers combined with data augmentation techniques for lithology identification. These approaches represent valuable progress, although they typically treat plane-polarized light (PPL) and cross-polarized light (XPL) images separately or reduce them to structured features, which may limit the ability to fully leverage the complementary optical information inherent in direct image integration. Similarly, studies such as those by^16^ and^17^ focus on QEMSCAN-derived mineralogical maps or drill core plug images, which, despite their analytical utility, rely on specialized and costly acquisition methods that are less common in routine reservoir characterization workflows.

Building upon these prior efforts, our study introduces a fully automated and modular pipeline that processes paired PPL and XPL images directly as raw inputs using twin convolutional neural networks, enabling a more integrated representation of petrographic features. Our workflow further incorporates YOLOv8-based region-of-interest detection, MobileNet feature extraction, MLP classification, and automated hyperparameter optimization with Optuna. Additionally, our classification system adopts a hierarchical structure, based on the scheme proposed by^19^ for the Pre-salt play carbonates, allowing for an explicit investigation of the impact of classification granularity, a dimension not yet systematically explored in this domain.

In the context of hydrocarbon exploration, developing ML-based techniques for lithological and petrographic analysis offers significant advantages. These methods provide cost-effective, rapid assessments of rock samples, which is particularly beneficial when dealing with large datasets from deep offshore reservoirs. Therefore, the aim of this study is not to replace the detailed manual interpretation conducted by experienced petrographers but to augment it with automated, high-throughput methods capable of pre-classifying and organizing extensive collections of petrographic images. By establishing this methodology, we aim to facilitate faster and more efficient interpretation workflows, addressing the growing demands of data-intensive environments such as the pre-salt reservoirs of the Santos Basin.

## Brazilian pre-salt carbonate reservoirs

**Fig. 1:**
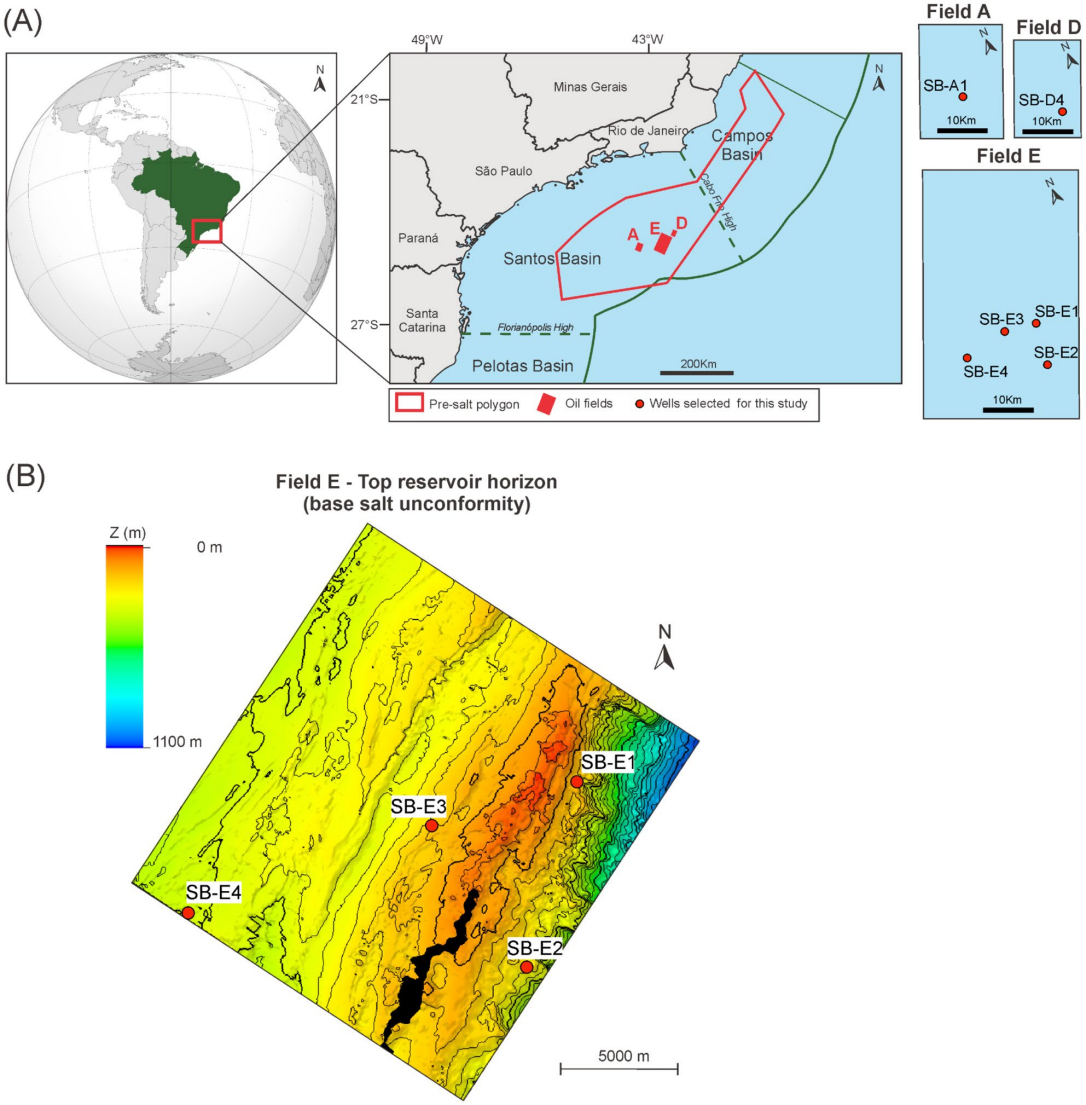
(**A**) Location of the Santos Basin, including the pre-salt polygon (red) and highlighting the three fields and six wells targeted in this work (map generated using QGIS version 3.36 http://qgis.org). (**B**) Top reservoir seismic horizon of Field E showing the location of the four wells in relation to the main NE-SW structural trend (map created using Petrel^TM ^version 2022 Schlumberger).

The Santos Basin is a passive-margin basin situated along the southeastern Brazilian continental margin, spanning approximately 350,000 km² between the states of Rio de Janeiro and Santa Catarina. Its northeastern boundary is defined by the Cabo Frio High, while the southwestern limit is marked by the Florianópolis Platform, which separates the basin from the Campos and Pelotas Basins, respectively (Fig. 1)^20^.

According to the National Petroleum Agency (ANP), in April of 2025, oil and gas production from Brazil’s pre-salt reservoirs reached 3,734 million barrels of oil equivalent per day (boe/d), representing an impressive 79.7% of the country’s total production. In the pre-salt section of the Santos Basin, the reservoirs are divided into two main formations dominated by carbonate rocks: the Barra Velha Formation (Aptian) and the Itapema Formation (Barremian-Aptian). The Barra Velha Formation, the main focus of this work, was deposited in sag-type basins (i.e., basins generated by thermal contraction and collapse and/or subsidence processes) during the post-rift phase, following the structurally controlled rift-phase basins where the Itapema Formation was deposited^21^.

**Fig. 2:**
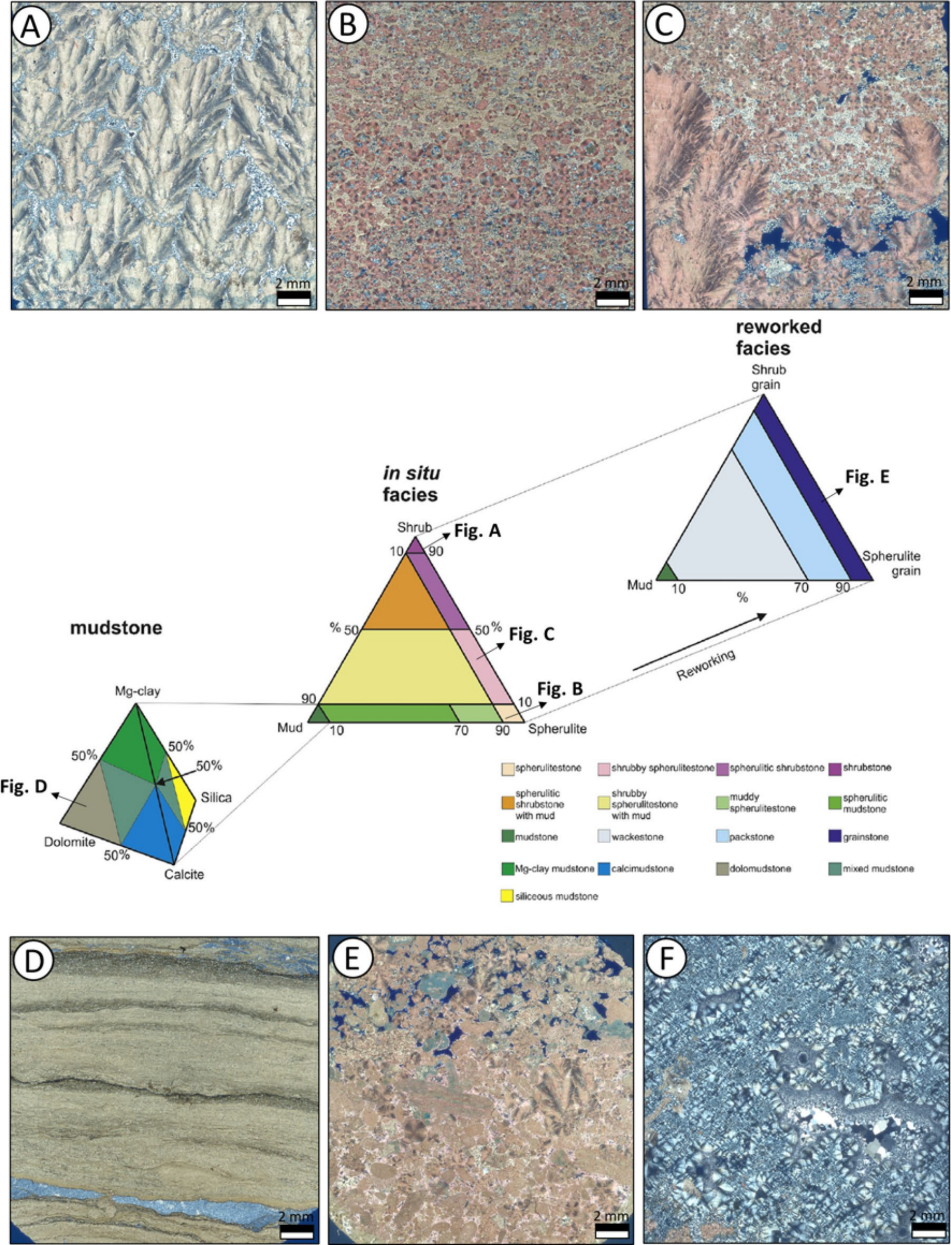
BVF classification scheme by^19^, illustrated with samples from this study’s dataset (XPL). Photomicrographs represent: (A) shrubstone, (**B**) spherulitestone, (**C**) shrubby spherulitestone, (**D**) dolomudstone, (**E**) grainstone, and (**F**) silicified carbonate, which is not included in the original diagram but was added to the classification due to its frequent occurrence in our dataset.

Currently, most studies addressing the characterization of facies from the sag stage of the South Atlantic basins mention a well-known and increasingly recognized set of facies, which mainly include: (1) facies dominated by shrubby carbonate elements or shrubs (Fig. 2 A) [e.g., shrubby boundstones^22^; framestones^23^; shrubstones^19^; (2) facies dominated by spherulites (Fig. 2 B, C) [e.g., spherulitic wackestones, packstones, and grainstones^22^; spherulites^19^; (3) mudstones and dolomudstones, sometimes rich in magnesium clays (Fig. 2 D)^23–25^; and (4) detrital carbonates formed by the reworking of the other facies (Fig. 2 E) [e.g., intraclastic grainstones and rudstones^22–24^.

Considering that these unusual carbonate fabrics of the Barra Velha Formation do not fit properly previous carbonate rock classifications (e.g^26^. and^27^)^19^, proposed a new facies classification based on sediment texture and composition (Fig. 2), considering the relative abundance of mud, calcite spherulites and fascicular calcite shrubs. This classification has become widely applied in the pre-salt carbonates (e.g^28–30^.).

Additionally, the facies mentioned above have complex carbonate and clay fabrics, whose primary composition can influence different diagenetic pathways^24^. Among the eodiagenetic processes, the alterations in the magnesian silicate matrices, such as dissolution and replacement by silica and dolomite, stand out^23,24,31^. On the other hand, deep burial alterations include the influence of hydrothermal fluids, which mainly promote dolomitization, silicification (Fig. 2F), and dissolution processes (e.g., hypogenic karstification), varying widely in intensity and influence on reservoir petrophysical properties^31^.

## Methodology

### Dataset

The database of this work includes 800 thin sections of the Barra Velha Formation of 6 wells from 3 different oilfields (here named as Field A, D, and E) of the Santos Basin (Fig. 1), SE Brazil. In an effort to compile a thin section database that is representative of the heterogeneity of the Barra Velha Formation, in addition to the selection of wells from different oil fields, for the case of Field E, wells were selected based on different paleotopographic contexts. Works such as^19^ and^32^ have evidenced the important facies trend associated with the distance from the paleotopographic structural highs. Therefore wells were selected from different parts of the field at different positions in relation to the main NE-SW structuring (Fig. 1 B).

Additionally, many of the wells included in this study have been the subject of prior detailed analyses, thereby enhancing the reliability of the manual interpretations. Detailed descriptions of well SB-A1 can be found in Basso et al.^33,34^ and^35^, while well SB-D4 was studied by^36^. Well SB-E1 was characterized in the works of^30^ and^37^.

The thin sections from all of these wells were described using conventional petrographic microscopy. High-resolution images, both in plane-polarized light (PPL) and cross-polarized light (XPL), were obtained using a 6400 dpi scanner, resulting in a total of 1600 images and 50.8 GB of data. All thin sections were stained with alizarin red and impregnated with blue epoxy resin to facilitate the identification of carbonate minerals and pore spaces, respectively.

### BVF facies classification

For this study, the thin section database was classified by an interpreter using three versions of the^19^ classification (Table 1): a complete version with 23 classes, an intermediate version with 9 classes, and a simplified version with 5 classes, representing a general facies upscaling. The aim was to evaluate the performance of the computational models across different levels of classification granularity.

The classification shown in Table 1 includes two additional classes beyond those in^19^: “Volcanic Grainstones” and “Silicified Carbonates.” These were added to account for the specific geological contexts of certain wells in this study. The “Silicified Carbonates” class includes samples where the primary depositional texture has been partially or completely obliterated due to heavy silicification. Meanwhile, the “Volcanic Grainstones” class encompasses impure grainstones rich in volcanic clasts, commonly found in the lower interval of the Barra Velha Formation in field E.

Additionally, in order to evaluate the significance of the different classifications schemes in relation to reservoir properties, the porosity of each thin section was quantified by image analysis. To quantify porosity, this study adopted the method developed by^38^, which evaluates the total optical porosity of thin sections impregnated with blue resin. This method employs the macro jPOR, a tool created by the authors, which can be applied to digital photomicrographs of thin sections using the ImageJ software.

**Table 1:**
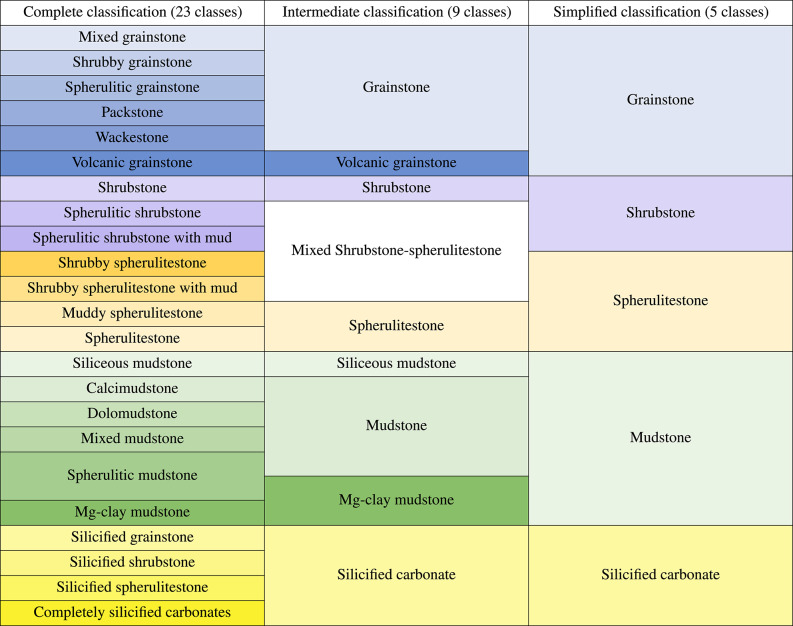
Three Barra Velha Formation classification schemes applied in this work (complete, intermediate and simplified).

### Computational model

**Table 2:** Summary of key components in the proposed workflow.

Component	Function in the workflow	Specific advantage	Challenge addressed
Paired PPL & XPL images	Use of raw dual-channel optical inputs	Captures complementary optical and textural features	Overcomes the loss of information typical in single-mode or feature-engineered approaches
YOLO v8	Automated Region of Interest (ROI) detection	Enables efficient, consistent cropping of relevant image areas	Removes subjective bias and reduces manual preprocessing effort
Deep convolutional vision models	Robust feature extraction	High capability of extracting features from images and preserving spatial correlation	Suitable for processing high-resolution petrographic images and extract relevant features
MLP	Final classification layer	Simple, effective method to combine learned features into predicted classes	Supports flexible adaptation to different classification schemes
Optuna	Automated hyperparameter tuning	Ensures optimal model configuration without extensive manual trial	Improves reproducibility and enhances model generalization

**Fig. 3:**
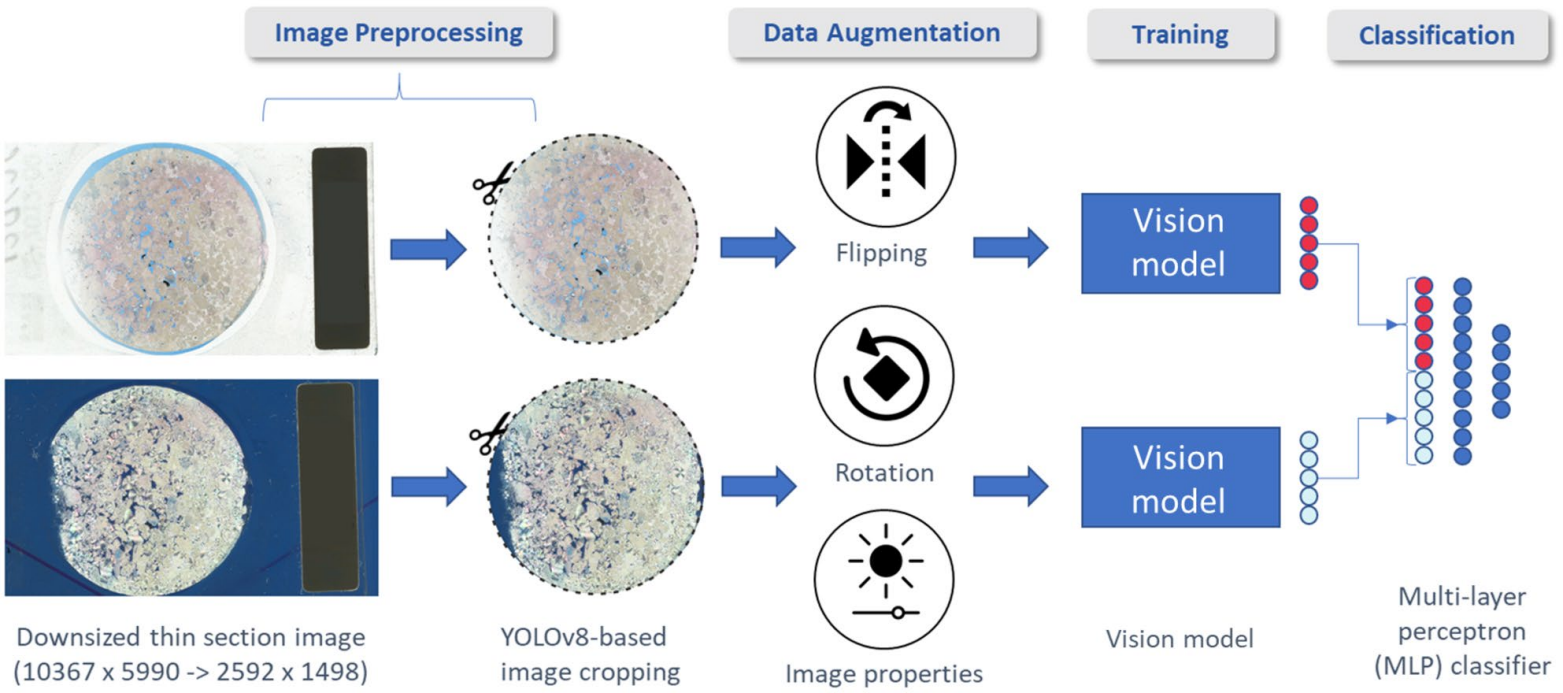
Computational model applied in this work based on twin vision models combined with a MLP classifier.

Different computational models with distinct configurations and parameters were tested before establishing the final model applied in this work. The workflow developed here (Fig. 3, Table 2) was based on the use of a computer vision model to extract features from the thin sections, one for each thin section light configuration, and classify the image pair. In order to find the best combination of hyperparameters for all parts in the workflow, we used the library called Optuna^39^, which is a hyperparameter search framework that allows an efficient search through the parameter space (Table 3). The search works by defining possible values for as many variables as needed in an objective function together with the code for the models. Following, we set a study with a certain number of trials and how the metrics should be read. During these trials, the framework selects one possible value to every variable set in the objective function, runs the code and returns a set of scores, which are used to evaluate how good the trial was. The advantage of using such libraries is the more elegant way to search through a parameter space that is not a simple brute force, such as a grid search, that can take much longer time to finish. The workflow for facies classification is divided in four main steps: (1) Image Preprocessing; (2) Data Augmentation; (3) Training; and (4) Classification.

The first step of the algorithm is responsible for preparing the thin section images to be fed into the model in a proper way. It starts with the reduction in size of the input image, due to limits on GPU memory. This reduction was made by a simple nearest neighbor interpolation followed by a filtering pass using a Gaussian filter ($\delta$ = 3/2) to avoid aliasing. Still in the preprocessing step, the second part is the cropping using the object detection model called YOLOv8 [The official documentation of the YOLOv8 can be found here], known for its speed and accuracy. The training process for the crop process was made by manually creating bounding boxes around the rock sample of a few thin sections and saving it as a new class specific to the rock sample. The next part is the model fine tuning, which took the already trained nano version of the model weights and further trained it for 150 epochs to properly add the new class to the model. Thereafter, the newly trained model was used to crop every thin section, both PPL and XPL, to extract the region of interest that were used to train the computer vision models.

For the feature extraction, we used a selection of computer vision models that have relatively small sizes and, hence, can deliver good results for such a small dataset. All models and pretrained weights were selected by their number of learnable parameters presented in the torchvision website [The list of available pretrained weights for classification models can be found here]. From all models available, we selected 8 models ranging from 3.5M to 11.7M parameters which were dynamically selected during Optuna trials. After, we filtered these models by their results and kept the first 3 best models for each classification version. After filtering, the models selected for the analysis were the EfficientNet (Tan and Le, 2019), MobileNet v3^40^, RegNet^41^, ResNet^42^ and ShuffleNet v2^43^, varying between versions of them related to their internal structure and number of parameter. All these models were created with low resource use and efficiency as the main objective, as many of them were created for mobile applications. This fact is interesting as they have a lower number of learnable parameters and, hence, would fit well enough with our dataset size. The differences, on the other hand, are more related to their internal mechanisms to achieve high speed and efficiency while keeping the size small, such as scaling techniques and special layers (channel shuffle, squeeze-and-excitation blocks, etc.).

During the training process, we added an augmentation step to artificially grow our dataset in an attempt to give more information to the model. The augmentation steps were random vertical and horizontal flips, random rotations from −30 to 30 degrees, and random alterations on the image properties, such as contrast, brightness, saturation and sharpness.

After the feature extraction, we used a Multi Layer Perceptron (MLP) to receive the features from the images and use them as input to classify them using one of the available classification versions. The number and size of layers of the MLP was also selected by Optuna during the study. The metrics used to evaluate the performance of each model were the F1-score and Accuracy. The F1-score is a metric based on the values of other two metrics, precision and recall, and calculated for each class. The equation of the F1-score is

1 $$\begin{aligned} Precision = \frac{TP}{TP+FP} \end{aligned}$$

2 $$\begin{aligned} Recall = \frac{TP}{TP+FN} \end{aligned}$$

3 $$\begin{aligned} F1-score = \frac{2.Precision.Recall}{Precision+Recall} = \frac{TP}{TP + \frac{1}{2}(FP+FN)} \end{aligned}$$

where TP means true positives, FP means false positives and FN means false negatives. After calculating the F1-score for every class in the classification, we calculated the simple average value between all classes, in a way to penalize when a class receives a low f1 score as the dataset is unbalanced. The accuracy is a metric that measures the overall correctness of the predictions. The equation to calculate the accuracy is:

4 $$\begin{aligned} Accuracy = \frac{TP+TN}{TP+TN+FP+FN} \end{aligned}$$

where TP means true positive, TN means true negative, FP means false positive and FN means false negatives. In our case, we opted to use the balanced accuracy to obtain a batter result considering the dataset imbalance. For that, we calculated the accuracy for each class and then averaged them into an overall balanced accuracy.

**Table 3:** Optuna hyperparameters space used for the architecture optimization process.

CNN models			Other parameters		
Model name	Variation	# of parameters	Feature extraction	# of filters	16 - 1024 (log)
EfficientNet	B0	5.3M		Pre-trained weights	Yes or No
MobileNet	v3 Small	2.5M	Classification head	# of filters	16 - 1024 (log)
MobileNet	v3 Large	5.5M		# of layers	2 - 12 (step of 2)
RegNet	Y 400mf	4.3M	Training	Learning rate	$10^{-5}$-$10^{-2}$(log)
RegNet	Y 800mf	6.4M		Batch size	^16^
RegNet	X 400mf	5.5M		Augmentation probability	0.3 - 0.7 (step of 0.1)
RegNet	X 800mf	7.3M			
ResNet	18	11.7M			
ShuffleNet	v2 x1.5	3.5M			
ShuffleNet	v2 x2.0	7.4M			

## Results and discussion

### Interpreter facies classification

**Fig. 4:**
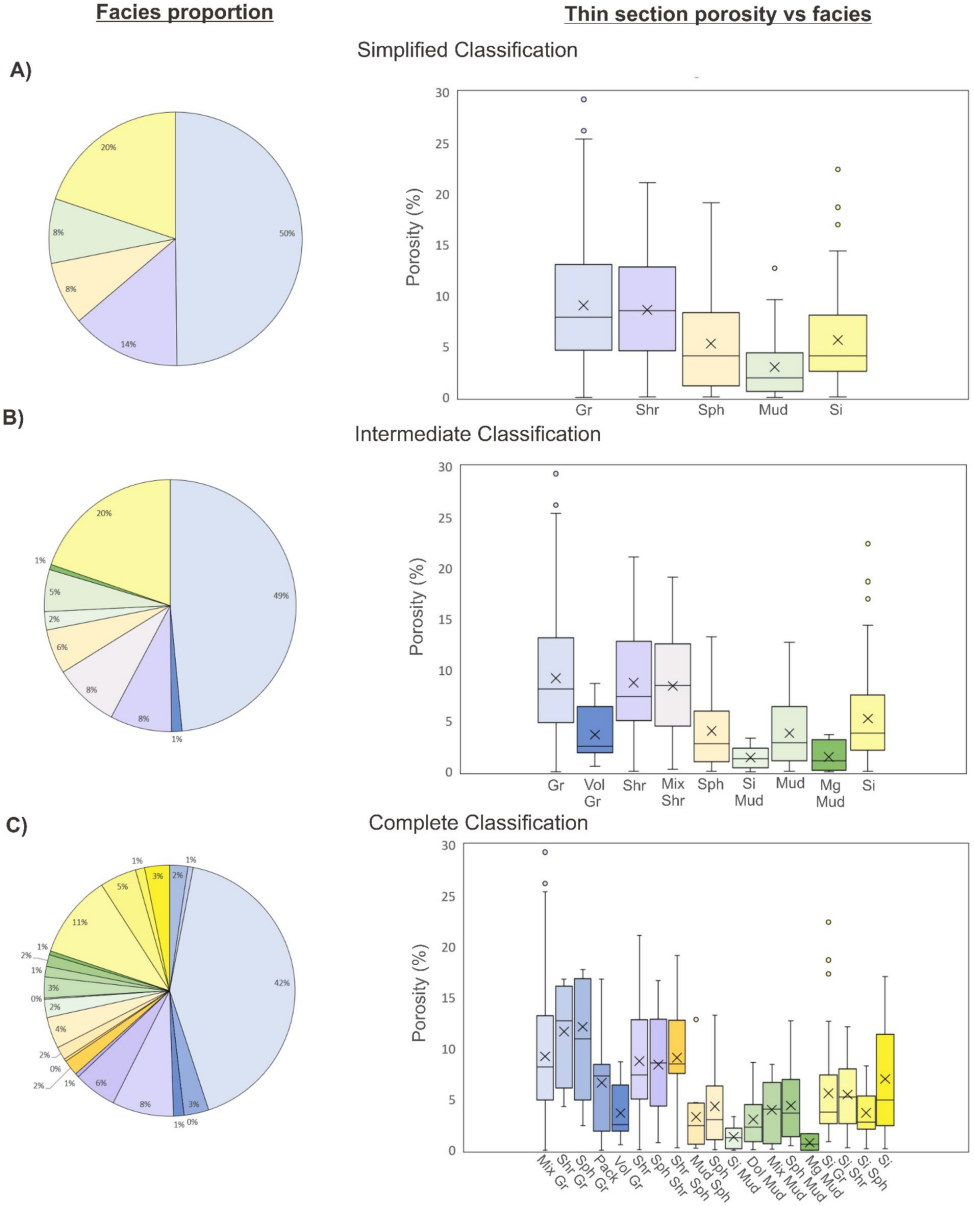
Pie charts of facies percentages resulting from the manual thin section classification (600 ts) and porosity per facies box, for the simplified (**A**), intermediate (**B**), and complete (**C**) classifications. The porosity values were extracted from the thin sections by image analysis technique. For color legend please refer to Table 1.

The manual classification of the thin sections, based on the different classification schemes, is presented in Fig. 4, illustrating the facies distribution across the dataset. The data distribution is clearly non-uniform. In the simplified classification, over 50% of the samples are classified as grainstones (light blue). This predominance persists in the intermediate (grainstone) and complete (mixed grainstone) classifications, though it decreases to 49% and 42%, respectively, due to the uneven subdivision of “subfacies” within the grainstone category.

The second most common group consists of silicified carbonates (yellow), which account for 20% of the total samples. Within this group, the silicified grainstone “subfacies” is predominant in the complete classification, representing more than half of the silicified samples. It is also important to note that in the complete classification scheme, several facies are underrepresented in the dataset, with nine classes comprising 1% or less of the thin sections.

Examining the behavior of the thin section estimated porosity for facies across the different classification schemes (Fig. 4) reveals that the simplified classification offers a reasonable upscaling, accurately reflecting the general porosity trends of the more complex classifications. However, this classification omits key information, since “subfacies” with distinct porosity behaviors are otherwise combined into broader categories.

The intermediate classification effectively separates pure grainstones and volcanic grainstones, which have very distinct petrophysical behavior, with pure grainstone showing higher reservoir potential^28,33^. Additionally, mixed shrubstone-spherulitestone facies are also individualized, showing higher porosity compared to pure spherulitestones. Similarly, different types of mudstones display distinct porosity behaviors that are not highlighted by the simplified classification.

Furthermore, in the complete classification, additional “subfacies” variations become evident, such as the higher porosity of completely silicified carbonates compared to the lower porosity of silicified spherulitestones. These differences in porosity within the same general group of “silicified carbonates” can be associated with different textural and genetic types of silica as discussed by^36^. The complete classification also brings to light more subtle facies differences, including the impact of preserved mud in certain facies, which can significantly affect porosity and reservoir quality.

In summary, both the intermediate and complete classifications reveal important geological and petrophysical information that is absent in the simplified classification, thereby justifying the evaluation and application of more complex classification schemes in the machine learning models developed in this study.

### Computational model analysis

**Fig. 5:**
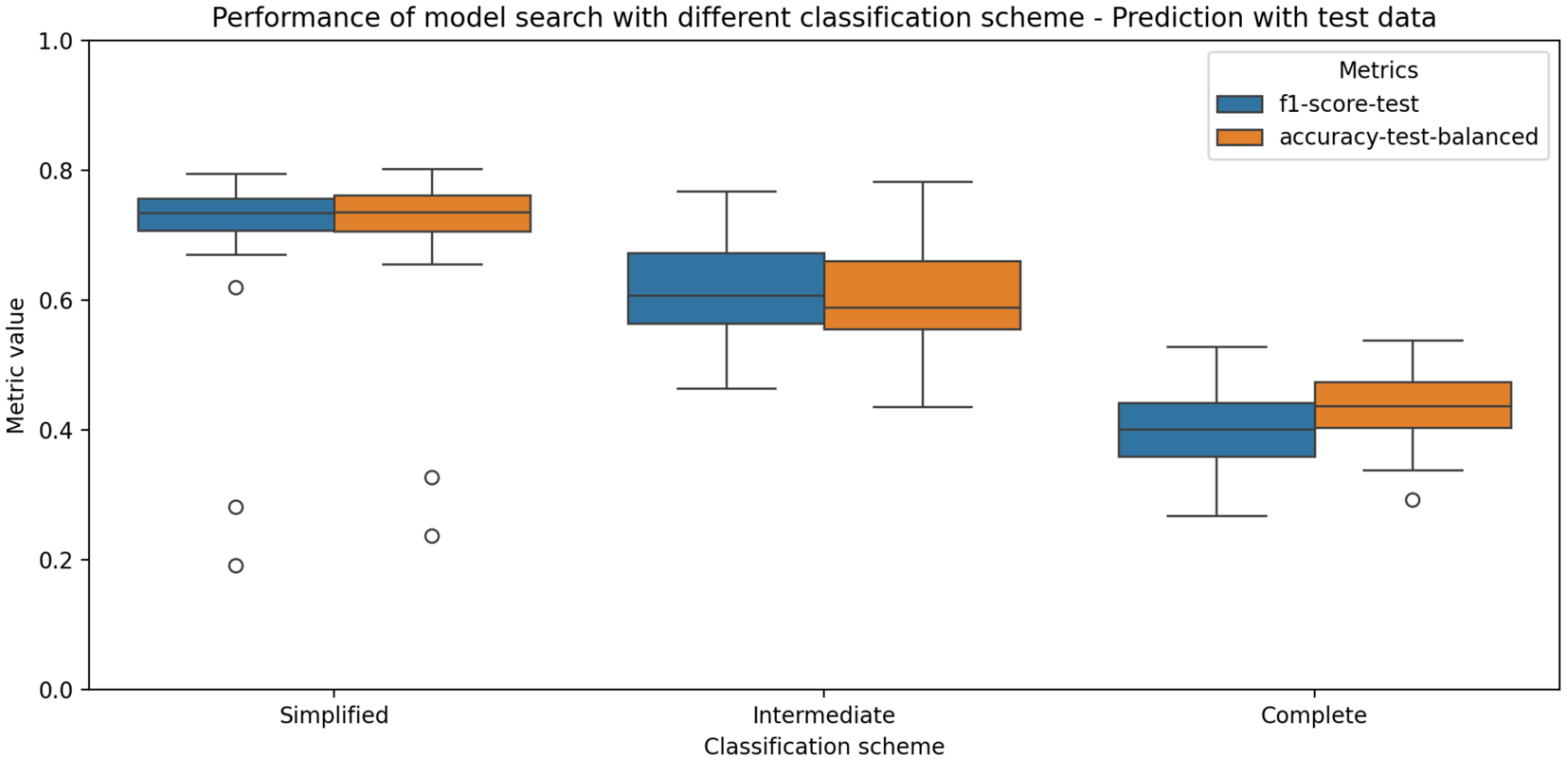
Summary of the computational models performance for the three classification schemes applied in this work. Note the general high metrics for the simplified and intermediate classification and lower metrics for the complete classification.

A total of 5 computational models were explored in this study, with their performance metrics, including F1-score and accuracy, summarized in Table 4 and illustrated in Figure 5. All models were evaluated across three classification schemes using only images from the validation set. A 70/30 train-test split was applied throughout the analysis.

Overall, the models performed well under the simplified and intermediate classification schemes, but there was a notable drop in metrics for the complete classification scheme (Fig. 5). The MobileNet v3 large model achieved the highest F1-score (0.795) and accuracy (0.802) for the simplified classification, while the RegNet X 400MF model yielded the best F1-score (0.768) for the intermediate classification. For the complete classification, the RegNet Y 400MF model attained the highest F1-score (0.528).

The general decline in accuracy and F1-score for the complete classification scheme is likely due to the non-uniform class distribution in the dataset, with many classes counting with just a minor number of samples for the model to be trained. This factor also affected the simplified and intermediate schemes, but had a more pronounced impact on the complete classification.

**Table 4:** Classification metrics for the best performing models for each classification version.

Classification version	Model name	F1-score	Accuracy
Simplified	Mobilenet v3 large	0.795	0.802
	Efficientnet B0	0.774	0.779
	Resnet18	0.769	0.764
Intermediate	Regnet X 400mf	0.768	0.731
	Regnet X 800mf	0.761	0.733
	Shufflenet v2 x2.0	0.756	0.751
Complete	Regnet Y 400mf	0.528	0.538
	Resnet18	0.493	0.516
	Shufflenet v2 x1.5	0.473	0.493

The simplified classification applied in this study is similar to the classification applied by^15^ for the Pre-salt carbonates, with the exception that here we have added the class “silicified carbonates”. The authors also used a comparable data set (570 thin sections) reaching an accuracy of 82.90%, similar to the accuracy presented by the model Mobilenet v3 large in this study, which was of 80.20%. Thus indicating the high potential for training with a lower number of classes.

**Fig. 6:**
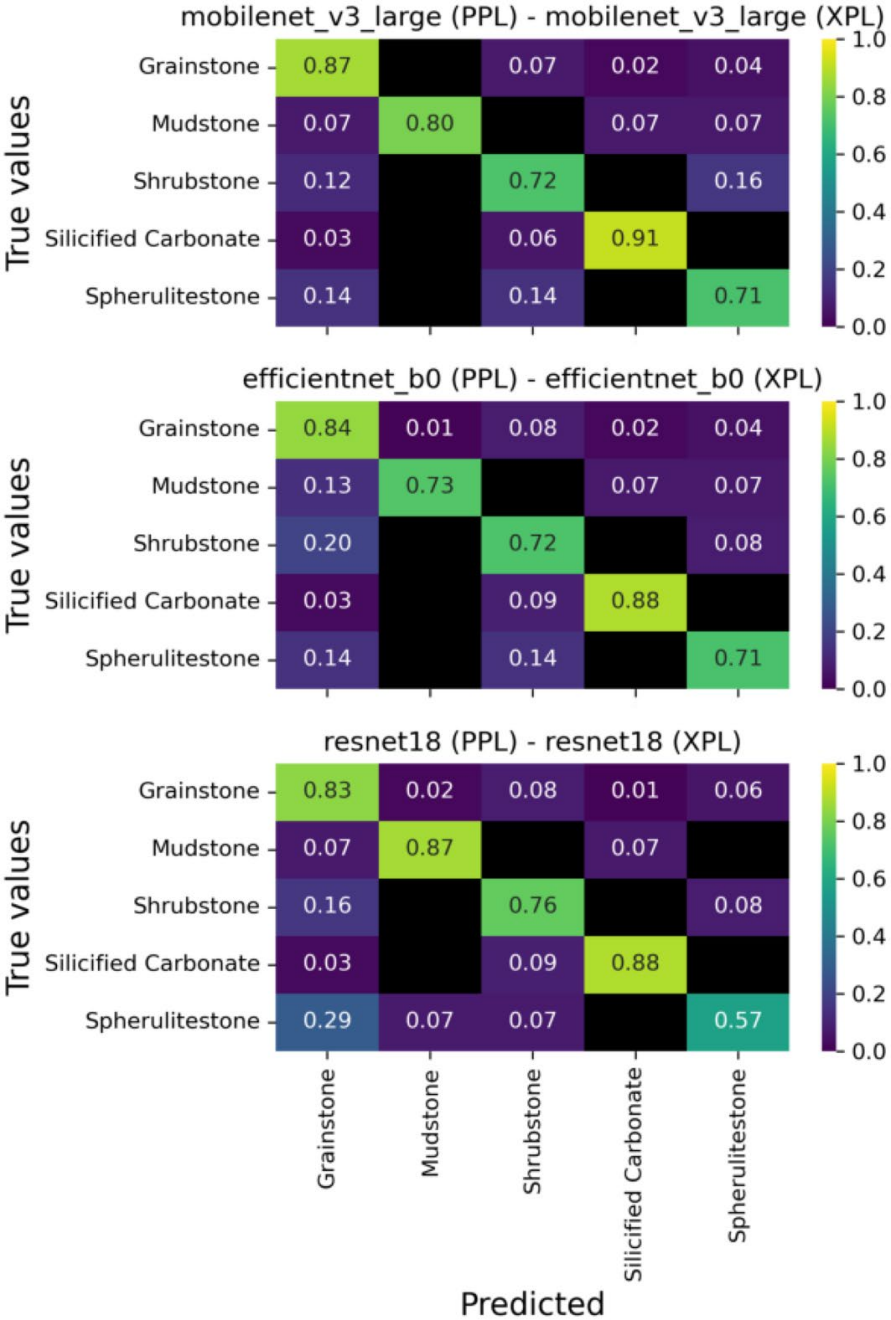
Confusion matrices of the three best performing models for the simplified classification scheme.

By examining the confusion matrix for the simplified classification (Fig. 6), we can see that all three of the best models delivered satisfactory predictions for all facies, with most scores above 0.7. However, despite these positive results, two main misclassifications are observed: (1) the model tends to misclassify shrubstones and spherulitestones as grainstones; and (2) shrubstones are often confused with spherulitestones, and vice versa.

**Fig. 7:**
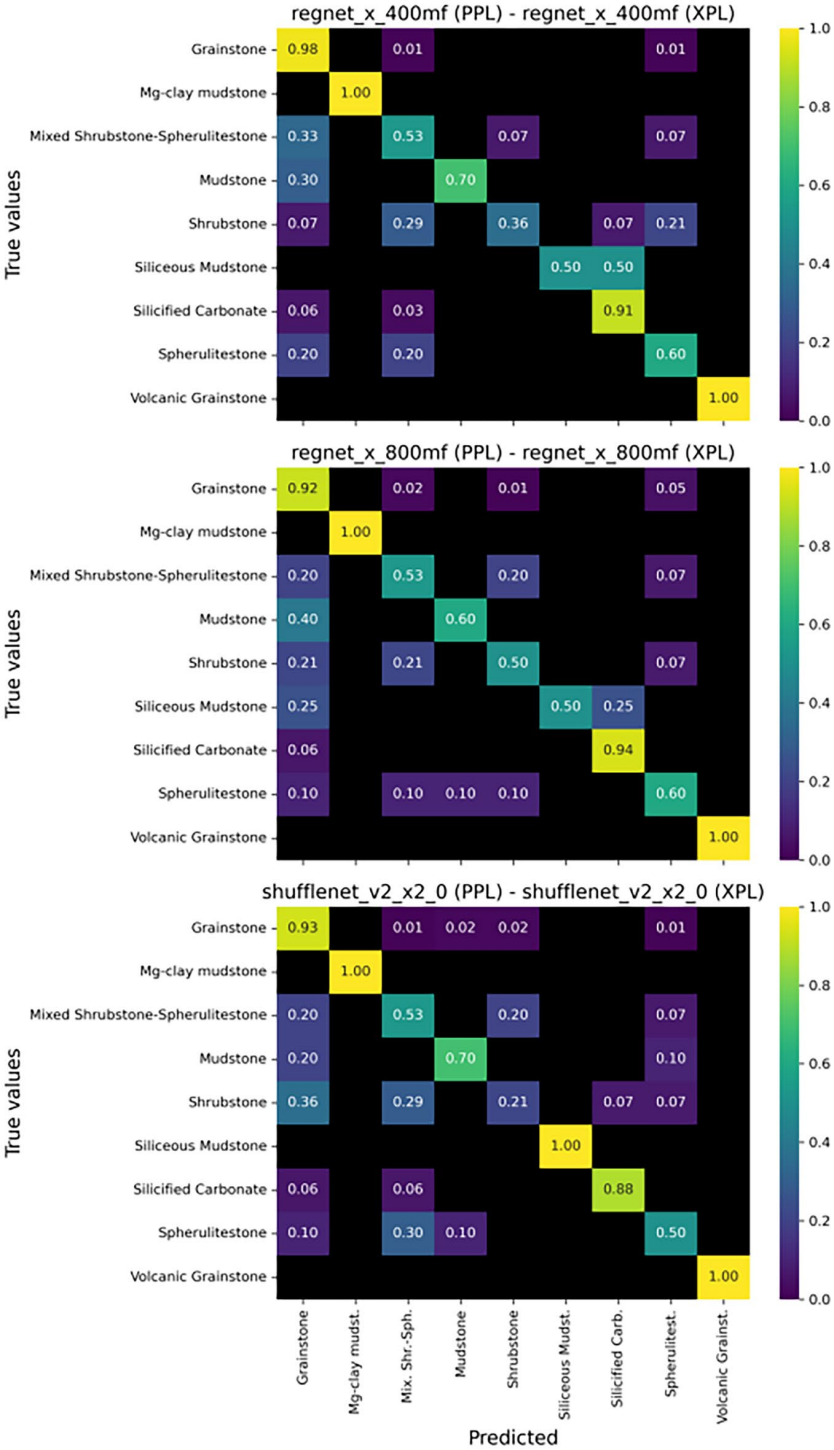
Confusion matrices of the three best performing models for the intermediate classification scheme.

Similarly, the models performed well in the intermediate classification (Fig. 7), with only a slight drop in metrics compared to the simplified classification. The same types of misclassifications occurred, now extending to the new classes but following the same patterns. The models tend to misclassify in situ lithologies as reworked carbonates (e.g., shrubstones and mixed shrubstone-spherulitestones as grainstones). Additionally, shrubstones are often misinterpreted as mixed shrubstone-spheruliticstones and spheruliticstones, and vice versa.

As noted by^15^, differentiating between in situ and reworked classes can be particularly challenging in the context of the Brazilian pre-salt carbonates, where grainstones often incorporate reworked fragments of various lithologies, including shrubstones and spherulitestones, reflecting complex autochthonous and allochthonous depositional processes. A key petrographic criterion for distinguishing between these deposits is the orientation of shrub structures: in situ shrubstones typically exhibit well-organized, aligned growth fabrics, indicative in-place precipitation. In contrast, reworked deposits, such as grainstones, often contain disoriented or fragmented shrub clasts, resulting from transport and redeposition under higher-energy conditions (Fig. 9A). However, this distinction is not always straightforward, as factors such as partial silicification, mechanical compaction, and heterogeneous diagenetic overprinting can obscure primary fabrics. Additionally, the scale and orientation captured in thin sections may not adequately represent the three-dimensional fabric, further complicating discrimination.

Moreover, distinguishing between shrubstones, mixed shrubstone-spherulitestone, and spherulitestone can be particularly challenging, as these lithologies often form a genetic and textural continuum, ranging from shrub-dominated fabrics to radial-fibrous spherulitic precipitates, with hybrid intermediate forms^22,33^ (Fig. 9C). This continuum arises from subtle variations in growth dynamics, precipitation kinetics, and environmental conditions, resulting in overlapping textural features that are difficult to differentiate consistently. Such complexity poses a significant challenge for automated classification models, which may struggle to capture these nuanced gradations within the carbonate fabric.

It is also important to highlight that the neural network has satisfactorily classified silicified carbonate samples within the simplified and intermediate classification, with only limited misclassifications by the models. These are particularly promising results, given the critical importance of accurately identifying silicified carbonates in the Brazilian pre-salt. Such silicified intervals are commonly associated with zones of enhanced fracture intensity and vuggy porosity, and reduced matrix porosity/permeability^36,44^. The higher concentration of vugs and fractures might induce excess-k layers which play a significant role in controlling reservoir quality and productivity^45^. The correct identification of silicified intervals has practical implications for drilling, by anticipating geomechanical heterogeneity and potential wellbore instability, and for production optimization, by informing completion strategies and reservoir modeling.

Another important advantage of the intermediate classification scheme is its ability to distinguish between pure grainstones and volcanic grainstones, which are rich in volcanic fragments derived from the economic basement (i.e. the Camboriú Formation). Petrographic image analysis reveals that these impure grainstones, containing altered volcanic clasts, typically exhibit significantly lower porosity due to the reduced primary pore space and the tendency for secondary mineral infill within intergranular pores. Notably, the model achieved an accuracy of 100% for this facies within the intermediate classification, underscoring the potential of the proposed workflow to effectively identify such petrophysically distinct lithotypes.

**Fig. 8:**
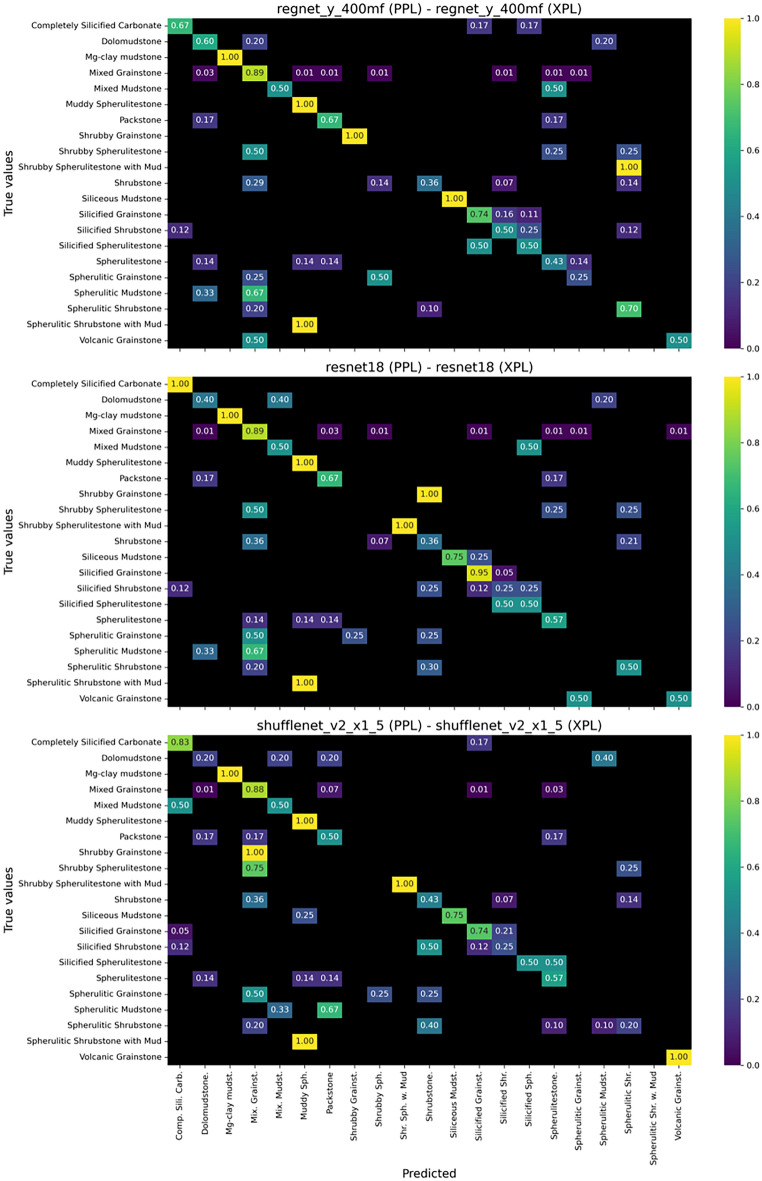
Confusion matrices of the three best performing models for the complete classification scheme.

Examining the confusion matrix for the complete classification (Fig. 8), we can see that while the models performed well for the recognition of certain facies (e.g., completely silicified carbonates, mixed grainstones, and shrubby grainstones), they struggled to correctly classify the totality samples from several other facies (e.g., shrubby spherulitestone and shrubby spherulitestone with mud). This is likely due to factors such as the smaller number of samples for these classes, as the dataset became more dispersed across additional categories, and the more subtle textural differences in this refined classification. It is important to note that not all misclassifications carry the same geological impact. For instance, misclassifying a high-permeability grainstone as a low-porosity spherulitestone may lead to more significant interpretive errors than confusions among genetically similar in situ facies. Future developments should incorporate domain-specific severity weighting in performance evaluation metrics to better reflect these geological priorities.

Finally, an important consideration arising from these results relates to the selection of classification granularity and its practical implications for petrographic database development. While the complete classification scheme provides the highest geological detail, the model’s lower predictive performance at this level, combined with the significant class imbalance, limits its practical utility for automated workflows. Conversely, the simplified scheme offers greater predictive accuracy but at the cost of losing key lithological distinctions relevant for reservoir characterization. The intermediate classification scheme, comprising nine classes, demonstrated the best balance between geological detail and model performance, making it the most suitable for the intended application of high-throughput petrographic image pre-classification.

**Fig. 9:**
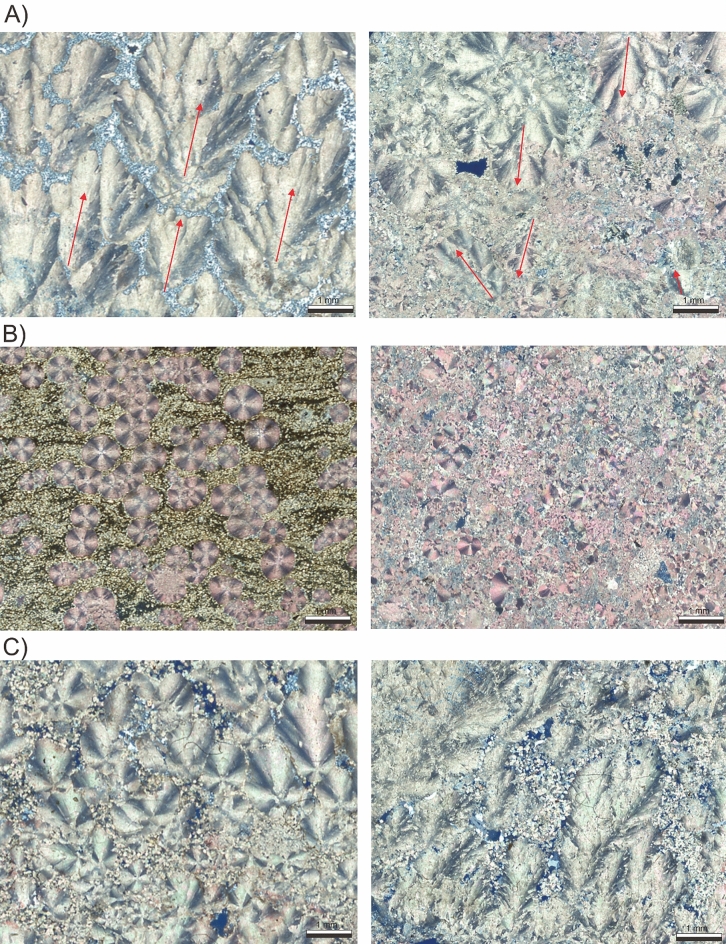
Examples of thin sections that are challenging to be classified by the CNN models. (**A**) Typical in situ shrubstones (left) vs grainstone formed by reworked shrubs (right), the red arrows indicate the growth direction which is evidence for reworking. (**B**) In situ spherulitestone (muddy sherulitestone) (left) vs reworked spherulitestone (right), note the presence of fragmented spherulites in the image on the right. (**C**) Spherulitestone with hybrid forms composed of asymmetrical spherulites and poorly developed shrubs (left) vs typical shrubstone (right).

### Independent blind testing

To evaluate the generalization capacity of the proposed workflow, an independent blind testing procedure was conducted using 200 thin sections (400 images) from well SB-E3, located in Field E. This well was not used in training, validation, or hyperparameter tuning, and provides sampling throughout the entire thickness of the Barra Velha Formation, making it especially suitable for assessing model performance across a complete stratigraphic profile.

**Fig. 10:**
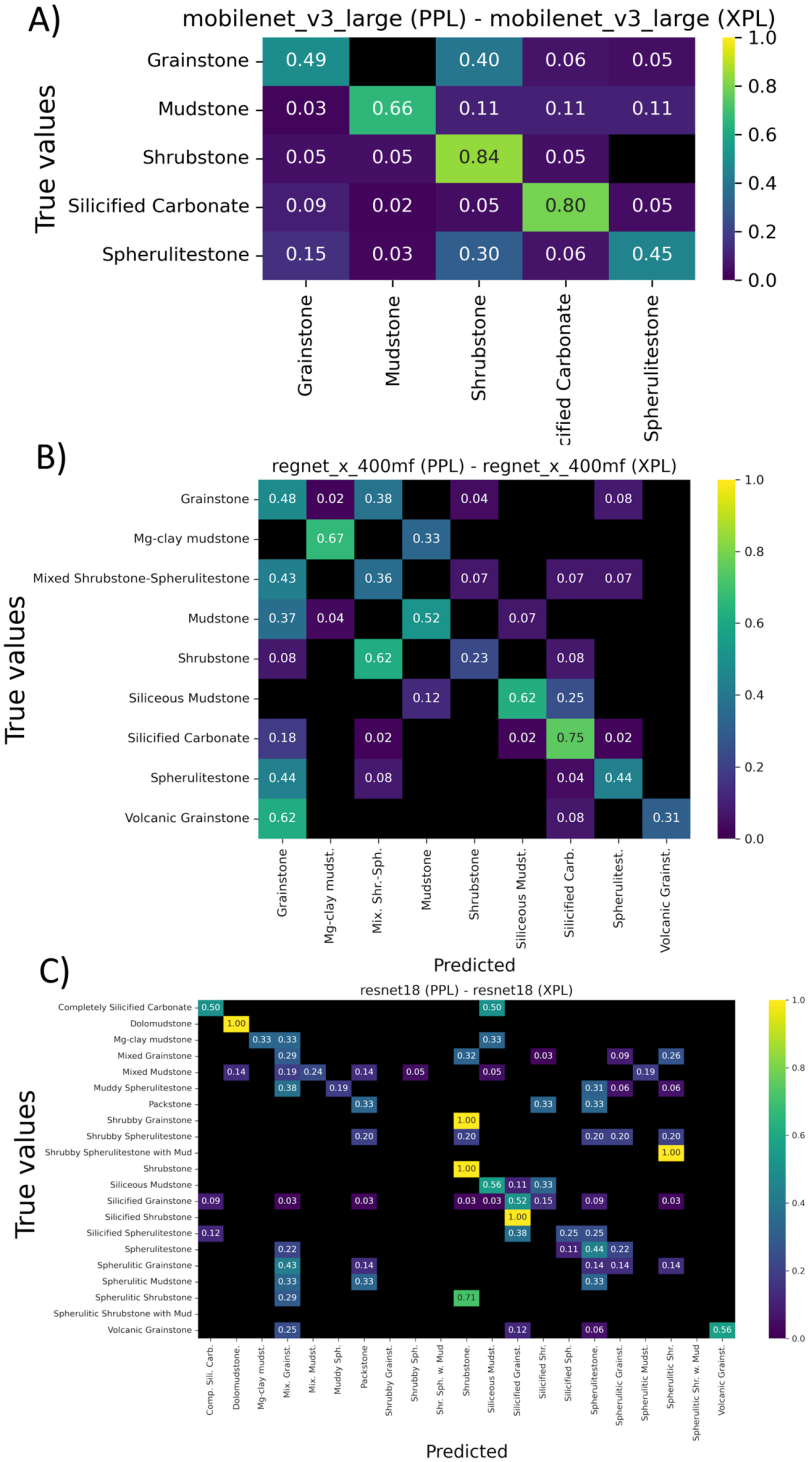
Confusion matrices for the best-performing models under blind testing using images from well SB-E3. (**A**) Simplified classification (model:MobileNet v3 large). (**B**) Intermediate classification (model: RegNet X 400MF)). (**C**) Complete classification (model: ResNet 18). The results reflect both recurrent misclassifications observed during training (e.g., shrubstones vs. grainstones) and additional confusion patterns specific to the blind test dataset, including new overlaps between volcanic grainstone and grainstone.

The performance results for the blind test are summarized in Table 5 and as confusion matrices in Figure 10. As expected, model accuracy and F1-score decreased with increasing classification complexity. The simplified scheme showed a mild drop compared to internal validation (Table 5), suggesting good robustness in low-complexity scenarios. The intermediate classification presented a moderate decline, but still delivered reasonable class separation, especially for key facies. In contrast, the complete scheme experienced a significant performance drop, with F1-scores below 0.29, reflecting the inherent difficulty of predicting a large number of visually similar facies in highly heterogeneous systems.

Despite lower overall metrics, the confusion matrices show that most misclassification patterns are consistent with those observed during validation, especially confusion between reworked and in situ facies. Additional errors emerged, particularly in the intermediate classification, where volcanic grainstones and mudstones were often misclassified as grainstones. These misclassification may be related to local depositional conditions (e.g. lesser proportion of reworked facies) and diagenetic characteristics (e.g. strong diagenetic overprint by dolomitization) of the SB-E3 well that were underrepresented in the training set. Importantly, even under blind testing, the models consistently identified silicified carbonates with high accuracy across classification schemes.

Lastly, part of the performance gap may be attributed to sampling. While the training dataset included multiple wells from distinct fields, the sampling was stratigraphically dispersed. In contrast, SB-E3 offers a continuous vertical section, introducing lithological transitions and microtextural features not fully captured during training. This underscores the importance of incorporating stratigraphically and diagenetically diverse examples in future datasets to improve model generalization.

**Table 5:** Model performance metrics in independent blind testing across classification levels.

Classification	Model	F1-score	Accuracy
Simplified	MobileNet v3 large	0.615	0.619
	Efficientnet B0	0.550	0.548
	ResNet 18	0.565	0.563
Intermediate	Regnet X 400mf	0.507	0.513
	Regnet X 800mf	0.477	0.508
	Shufflenet v2 x2.0	0.495	0.518
Complete	Regnet Y 400mf	0.209	0.335
	ResNet 18	0.286	0.371
	Shufflenet v2 x1.5	0.194	0.279

### Limitations and mitigation strategies

CNN-based models are inherently susceptible to overfitting, particularly when trained on datasets of moderate size with high intra-class variability, as is typical in heterogeneous carbonate systems. As in previous studies applying machine learning to pre-salt petrographic datasets (e.g^15^^18^), the moderate size and complexity of the data present challenges related to overfitting. To mitigate this risk, we implemented data augmentation techniques, including rotations, flips, and scaling, to increase the diversity of the training data and reduce overfitting tendencies. Additionally, we employed CNN architectures with varying levels of complexity. The inclusion of lightweight models was motivated by their reduced risk of overfitting in scenarios with limited training data, due to their smaller number of parameters.

Beyond model architecture, the performance of image-based models is also influenced by image quality and consistency. While all images in this study were acquired using standardized protocols at 6,400 dpi, variations in sample preparation, staining, and lighting conditions could still affect model performance. Ensuring consistent imaging protocols remains essential for the successful application of such models in practice.

Another important consideration is model interpretability. The interpretability of CNNs remains a known challenge, as these models often provide limited direct insight into the specific features guiding their predictions. Although interpretability was beyond the scope of this study, future research could incorporate explainable AI (XAI) techniques to improve transparency and foster greater confidence in model decisions.

The confusion matrices revealed consistent misclassification patterns, particularly between shrubstones, spherulitestones and grainstone made of these former lithologies. These errors likely arise from genuine geological ambiguities, including gradational textural boundaries, as well as the transitional nature of some depositional environments in the Barra Velha Formation. Future work could incorporate domain-specific features, such as grain orientation, size distributions, or mineralogical attributes, that may provide more discriminative power between visually similar classes. The integration of multi-modal data, including geochemical or mineralogical analyses, also represents a promising way for enhancing classification accuracy.

Finally, emerging architectures such as Vision Transformers (ViTs) represent a promising direction for future research. Their ability to model long-range spatial dependencies and capture subtle textural variations may be particularly valuable for petrographic image classification, especially in complex carbonate systems. Although not explored in the present study, these models may perform well when supported by larger and more balanced datasets, and their potential merits further investigation.

## Conclusion

Deep learning neural networks and computational vision techniques can be satisfactorily used to classify lithologies in thin sections of highly complex carbonate sedimentary systems. The models were able to process large datasets and identify patterns, offering a promising approach for facies prediction. This study yielded the following conclusions:

- The study highlights the variation in model performance across classification granularity. The simplified and intermediate classifications achieved high accuracy and F1-scores during validation, while the complete classification showed reduced performance, likely due to the increased number of underrepresented classes and overlapping features. The intermediate classification with a total of nine classes provided the most favorable trade-off between geological detail and predictive consistency.
- The independent blind test, conducted on a well excluded from training and validation, served as a realistic stress test of generalization. The test demonstrated that the models retain good generalization capacity in the simplified classification scheme, and show promising potential in the intermediate scheme, particularly if supported by a more representative and stratigraphically comprehensive training dataset.
- The models were particularly successful in identifying silicified carbonates in both validation and testing datasets, which are crucial for understanding silicification processes and its impact in the dual-porosity/dual-permeability reservoir properties of the Brazilian Pre-Salt play.

These findings demonstrate that the proposed workflow is especially well-suited for automated pre-classification and data organization, providing a valuable starting point for expert interpretation. While challenges remain in achieving high-resolution classifications, the approach offers a scalable and adaptable framework, one that stands to improve as more stratigraphically comprehensive training data become available. Also, the method shows strong potential for operational workflows requiring efficient handling of large petrographic datasets.

While applied here to thin sections of carbonate rocks, the workflow and classification strategy are broadly applicable to both sedimentary and non-sedimentary systems. Such approaches hold strong potential for advancing petrographic analysis across a range of geological contexts in academic and industrial settings.

## Data Availability

The datasets generated and analyzed during the current study are not publicly available due to internal data management policies, but are available from the corresponding author upon reasonable request.

## Code availability

The repository can be accessed at the following link: https://github.com/Energy-Production-Innovation-Center/thin-section-classifier.
